# Capsaicin-sensitive sensory nerves exert complex regulatory functions in the serum-transfer mouse model of autoimmune arthritis

**DOI:** 10.1016/j.bbi.2014.12.012

**Published:** 2015-03

**Authors:** Éva Borbély, Bálint Botz, Kata Bölcskei, Tibor Kenyér, László Kereskai, Tamás Kiss, János Szolcsányi, Erika Pintér, Janka Zsófia Csepregi, Attila Mócsai, Zsuzsanna Helyes

**Affiliations:** a Department of Pharmacology and Pharmacotherapy, University of Pécs, Medical School, Pécs, Hungary; b János Szentágothai Research Centre, Molecular Pharmacology Research Team, University of Pécs, Pécs, Hungary; c Centre for Neuroscience, University of Pécs, Medical School, Pécs, Hungary; d Department of Pathology, University of Pécs, Medical School, Pécs, Hungary; e Department of Physiology, and MTA-SE “Lendület” Inflammation Physiology Research Group, Semmelweis University, School of Medicine, Budapest, Hungary; f PharmInVivo Ltd, Pécs, Hungary; g MTA-PTE NAP B Pain Research Group, Hungary

**Keywords:** Capsaicin-sensitive sensory nerves, Pain, Inflammation, Somatostatin, Matrix-metalloproteinase

## Abstract

**Objective:**

The K/BxN serum-transfer arthritis is a widely-used translational mouse model of rheumatoid arthritis, in which the immunological components have thoroughly been investigated. In contrast, little is known about the role of sensory neural factors and the complexity of neuro–immune interactions. Therefore, we analyzed the involvement of capsaicin-sensitive peptidergic sensory nerves in autoantibody-induced arthritis with integrative methodology.

**Methods:**

Arthritogenic K/BxN or control serum was injected to non-pretreated mice or resiniferatoxin (RTX)-pretreated animals where capsaicin-sensitive nerves were inactivated. Edema, touch sensitivity, noxious heat threshold, joint function, body weight and clinical arthritis severity scores were determined repeatedly throughout two weeks. Micro-CT and *in vivo* optical imaging to determine matrix-metalloproteinase (MMP) and neutrophil-derived myeloperoxidase (MPO) activities, semiquantitative histopathological scoring and radioimmunoassay to measure somatostatin in the joint homogenates were also performed.

**Results:**

In RTX-pretreated mice, the autoantibody-induced joint swelling, arthritis severity score, MMP and MPO activities, as well as histopathological alterations were significantly greater compared to non-pretreated animals. Self-control quantification of the bone mass revealed decreased values in intact female mice, but significantly greater arthritis-induced pathological bone formation after RTX-pretreatment. In contrast, mechanical hyperalgesia from day 10 was smaller after inactivating capsaicin-sensitive afferents. Although thermal hyperalgesia did not develop, noxious heat threshold was significantly higher following RTX pretreatment. Somatostatin-like immunoreactivity elevated in the tibiotarsal joints in non-pretreated, which was significantly less in RTX-pretreated mice.

**Conclusions:**

Although capsaicin-sensitive sensory nerves mediate mechanical hyperalgesia in the later phase of autoantibody-induced chronic arthritis, they play important anti-inflammatory roles at least partially through somatostatin release.

## Highlights

• Capsaicin-sensitive sensory nerves are protective against autoimmune arthritis.
• Desensitization of these fibers increase immune cell activation and edema.
• Sensory denervation enhances ROS production, MMP activity and arthritic changes.
• Late mechanical hyperalgesia is decreased after destroying these sensory nerves.

## 1 Introduction

Rheumatoid arthritis (RA) is chronic autoimmune disease characterized by the destruction and deformation of the joints leading to persistent pain, movement disability and decreased life quality. It is a great public health problem worldwide due to its high incidence and prevalence, unsatisfactory therapeutic outcomes and unfavorable life expectancy (Kourilovitch et al., 2014; Jones et al., 2003). Despite promising novel drugs introduced recently in its pharmacotherapy, we still have to cope with several resistant cases and severe drug-induced adverse effects (Schett and Gravallese, 2012; Alarcón, 2000). Although our knowledge about the immunological aspects of the pathophysiological mechanisms has extensively increased in the last decade, the regulatory role of sensory nerves and the complexity of neuro–immune interactions in this condition are still not understood (Levine et al., 2006; Pongratz and Straub, 2010; Meinel et al., 2013; Stangenberg et al., 2014).

Capsaicin-sensitive peptidergic sensory nerves densely innervate the joint capsule and the synovium, which do not only mediate pain (classical afferent function), but also play an important role in inflammation via sensory neuropeptide release (efferent function). The Transient Receptor Potential Vanilloid 1 (TRPV1) non-selective cation channel located on these nerves is activated and sensitized by a variety of exogenous irritants, such as capsaicin, and resiniferatoxin (RTX), as well as endogenous molecules like protons, bradykinin, prostanoids, tumor-necrosis factor-α, nerve growth factor, gasotransmitters or lipid peroxidase products (Yoo et al., 2014). Many of these are crucial participants of inflammatory processes in RA. As a result of activation of the capsaicin-sensitive nerve terminals, sensory neuropeptides are released, such as the proinflammatory tachykinins (substance P, neurokinin A) and calcitonin-gene related peptide (CGRP) responsible for vasodilation and inflammatory cell recruitment (neurogenic inflammation) (Maggi, 1995; Szolcsanyi, 1996), as well as somatostatin, which is a potent antiinflammatory and antinociceptive agent. We have provided several lines of evidence in a variety of inflammation models that the overall role of these fibers depends on the functional significances of the simultaneously released pro- and antiinflammatory peptides in the respective pathophysiological processes (Pintér et al., 2014). We have also shown that sensory nerve-derived somatostatin is an important endogenous inhibitor in the adjuvant-induced arthritis model of the rat (Helyes et al., 2004).

The pathophysiological relevance of these peptides in humans is beyond doubt, since increased proinflammatory and decreased antiinflammatory neuropeptide levels have been demonstrated in the serum and/or synovial fluid of RA patients (Anichini et al., 1997; Larsson et al., 1991; Denko and Malemud, 2004).

Investigating rheumatoid arthritis mechanisms in animals is difficult; therefore there are many different rodent models which can more or less mimic the main symptoms of the disease (Bevaart et al., 2010; Zhang et al., 2012; Boettger et al., 2010). The K/BxN serum-transfer arthritis is a widely-used translational mouse model of RA, it shares a lot of similarities to the human disease, e.g. swelling of distal joints of all the paws with erosive synovitis, caused by the activation of neutrophils, macrophages, complement system which play a pivotal role in the induction and maintenance of arthritis (Kouskoff et al., 1996; Korganow et al., 1999; Fukushima et al., 2010). The immunological components of this model have thoroughly been investigated (Németh et al., 2010; Hickman-Brecks et al., 2011), but nothing is known about the role of sensory neural factors and the complexity of neuro–immune interactions. Therefore, we analyzed the involvement of capsaicin-sensitive peptidergic sensory nerves in autoantibody-induced arthritis with integrative methodology after the functional impairment of these fibers with high dose RTX pretreatment (desensitization).

## 2 Material and methods

### 2.1 Ethics statement

Experiments were carried out according to the 1998/XXVIII Act of the Hungarian Parliament on Animal Protection and Consideration Decree of Scientific Procedures of Animal Experiments (243/1988), complied with the recommendations of IASP, and approved by the Ethics Committee on Animal Research of University of Pécs (licence: BA 02/2000-2/2012).

### 2.2 Experimental animals

Male and female C57Bl/6 mice (10–12-week-old; 25–30 g) bred and kept in the Laboratory Animal House of the Department of Pharmacology and Pharmacotherapy of the University of Pécs at 24–25 °C under a 12-h light–dark cycle were used in all studies. Standard mouse chow and water were provided *ad libitum*.

### 2.3 Resiniferatoxin pretreatment

Pretreatment with the ultrapotent TRPV1 agonist resiniferatoxin (RTX, Sigma–Aldrich; 30, 70, 100 μg/kg s.c. on 3 consecutive days) leads to long-lasting defunctionalization of capsaicin-sensitive nerves (desensitization) (Szolcsanyi et al., 1990). Two weeks later the success of the pretreatment was verified by the lack of eye-wiping after capsaicin drops (50 μl, 0.1%) (Helyes et al., 2004).

### 2.4 Induction of arthritis

Chronic arthritis of male and female C57Bl/6 mice was induced by intraperitoneal (i.p.) injection of 150–150 μl of K/BxN serum on the days 0 and 3. Control groups of intact animals were treated with BxN (not arthritogenic/control) serum following the same protocol.

### 2.5 Assessment of arthritis severity and paw edema

Hind paw volume was measured by plethysmometry (Ugo Basile 7140, Comerio, Italy) (Helyes et al., 2004; Szabó et al., 2005). Arthritic changes were semiquantitatively scored using a grading scale of 0–10 (0–0.5: no change, 10: maximal inflammation) by evaluating edema and hyperemia (Németh et al., 2010). Volumes and scores were assessed before serum injection and every day during the 2-week period.

### 2.6 Measurement of mechanical and thermal hyperalgesia

Mechanonociceptive threshold of the paw was determined by dynamic plantar aesthesiometry (Ugo Basile 37400, Comerio, Italy) before and after serum administration. Mechanical hyperalgesia was expressed as % of initial, control mechanonociceptive thresholds (Helyes et al., 2004; Szabó et al., 2005). The thermonociceptive threshold of the paw was determined on increasing temperature hot plate (IITC Life Sciences, Woodland Hills, CA, USA) by nocifensive reactions (lifting, licking, shaking) or reaching the maximum value (53 °C) (Almási et al., 2003).

### 2.7 Assessment of joint function (grid test)

An easy and reproducible method to determine grasping ability correlating with joint function. Mice were placed on a horizontal wire-grid, then it was turned over and the latency to fall was determined (Németh et al., 2010).

### 2.8 Measurement of arthritis induced weight loss

As a typical sign of systemic effect of arthritis, mice lost weight after serum administration. Weight measurements were performed daily and weight loss was expressed in % of control values.

### 2.9 *In vivo* bioluminescence imaging of myeloperoxidase-activity

Luminol bioluminescence (BLI; 5-amino-2,3-dihydro-1,4-phthalazine-dione) correlates with neutrophil myeloperoxidase activity in arthritis *in vivo* (Chen et al., 2004; Gross et al., 2009). Na–luminol (150 mg/kg i.p., Sigma–Aldrich) dissolved in PBS (20 mg/ml) was injected on days 0, 2 and 6. Images were acquired 10 min later with IVIS Lumina II (PerkinElmer, Waltham, USA; 60 s acquisition, F/Stop = 1, Binning = 8). Identical Regions of Interests (ROIs) were applied around the ankles and luminescence was expressed as total radiance (total photon flux/s).

### 2.10 *In vivo* fluorescence imaging of matrix-metalloproteinase activity

Matrix-metalloproteinase (MMP) activity was assessed *in vivo* on days 5 and 8 using MMPSense680 (PerkinElmer), an activatable fluorescent imaging probe for MMP-2, -3, -9 and -13 according to the manufacturer’s instructions (2 nmol/subject i.v.). Measurements were performed with the FMT 2000 fluorescence molecular tomography system 24 h later (PerkinElmer). Three-dimensional reconstructions of the ankles were made, isocontour ROIs were applied, and MMP was expressed as pmol fluorophore.

### 2.11 *In vivo* micro-computed tomography (micro-CT) analysis of the periarticular bone structure

The right tibiotarsal joints were repeatedly (days 0, 7, 14) scanned by SkyScan 1176 *in vivo* micro-CT (Bruker, Kontich, Belgium) with 17.5 μm voxel size. Changes of bone structure were evaluated by CT Analyser® software. Standard size ROIs were applied around the periarticular tibia and fibula regions, and around the tibiotarsal and tarsometatarsal joints. Bone volume (μm^3^) was quantified and expressed as a percentage of the total ROI volume.

### 2.12 Histological processing and assessment of joint inflammation

Ankle joints excised on day 14 were fixed, decalcified and dehydrated, embedded in paraffin, sectioned (3–5 μm) (Helyes et al., 2004; Szabó et al., 2005) and stained with hematoxylin–eosin or Safranin O for detecting collagen deposition and fibroblasts. Histopathological changes were scored by a pathologist blinded from the study on the basis of (1) areolar tissue size and mononuclear cell infiltration, (2) synovial cell proliferation, (3) fibroblast number and collagen deposition to create composite arthritis scores (between 0 and 9) (Botz et al., 2014).

### 2.13 Determination of somatostatin-like immunoreactivity (SOM-LI) in tissue homogenates

Separate groups of mice were sacrificed in deep anesthesia on day 10 when both swelling and hyperalgesia were remarkable. The tibiotarsal joints were homogenized in a solution containing 20 mM KH_2_PO_4_ and K_2_HPO_4_ for 2 min at 24,000 rpm with Miccra D-9 Digitronic device (Art-moderne Laborteknik, Germany). Homogenates were centrifuged for 10 min at 4000 rpm afterwards for 15 min at 10,000 rpm and the supernatants were collected for SOM-LI determination with a specific and sensitive radioimmunoassay (RIA) (Németh et al., 1996).

### 2.14 Statistical analysis

All functional, histopathological and CT results were presented and evaluated separately for male and female mice, data points represent means ± SEM. Hyperalgesia, edema and weight loss were evaluated by repeated measures two-way analysis of variance (ANOVA) + Bonferroni’s modified *t*-test, semiquantitative clinical and composite histopathological scores by non-parametric Kruskal–Wallis test + Dunn’s post-test, micro-CT results by two-way ANOVA + Dunnett and Tukey post-tests to evaluate the time-dependent self-control changes and the different groups, respectively. Bioluminescence and fluorescence imaging, as well as somatostatin-LI were analyzed by Student’s *t*-test for unpaired comparisons. *p* < 0.05 was considered to be significant.

## 3 Results

### 3.1 Increased joint edema after desensitization of capsaicin-sensitive sensory nerves

**Fig. 1:**
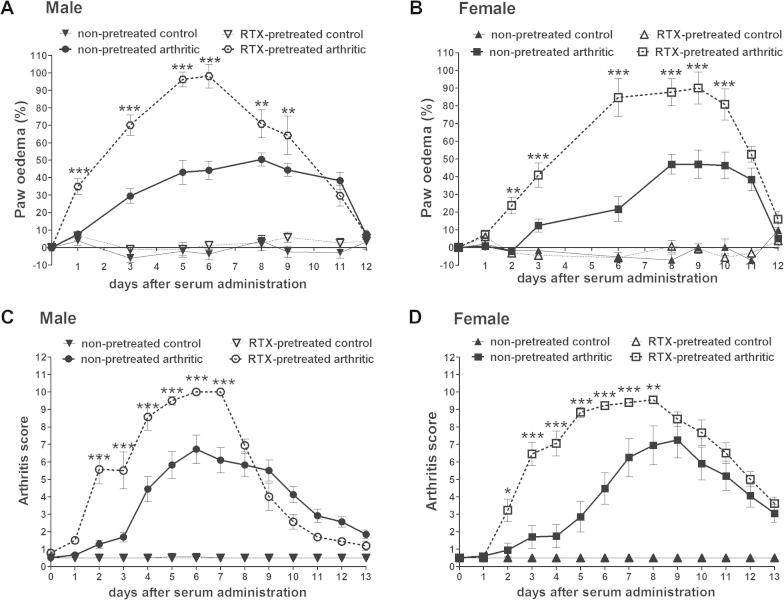
Serum-induced edema and arthritis score throughout the 2 weeks experimental period. Data points represent the percentage increase of the paw volume of male (A) and female (B) mice compared to the initial control values and the absolute values of arthritis scores for male (C) and female animals (D) (*n* = 4–5/non-inflamed groups, *n* = 6–8/arthritic groups; ^∗^*p* < 0.05, ^∗∗^*p* < 0.01, ^∗∗∗^*p* < 0.001 vs. non-pretreated; two-way ANOVA + Bonferroni’s modified *t*-test).

In non-pretreated arthritic mice an approximately 45% edema developed in both males and females, which was maintained till the end (day 11) of the experiment. In RTX-desensitized arthritic animals this swelling was significantly higher in both genders during the whole study with a maximum of 90–95% (Fig. 1A and B). Similarly, arthritis scores reached a maximum of 7 in mice without pretreatment and 9 in RTX-pretreated animals showing that the significant increase in paw volume was visible on all limbs between days 2 and 7–8 in male female mice, respectively (Fig. 1C and D).

### 3.2 Attenuated late mechanical hyperalgesia in RTX-desensitized mice

**Fig. 2:**
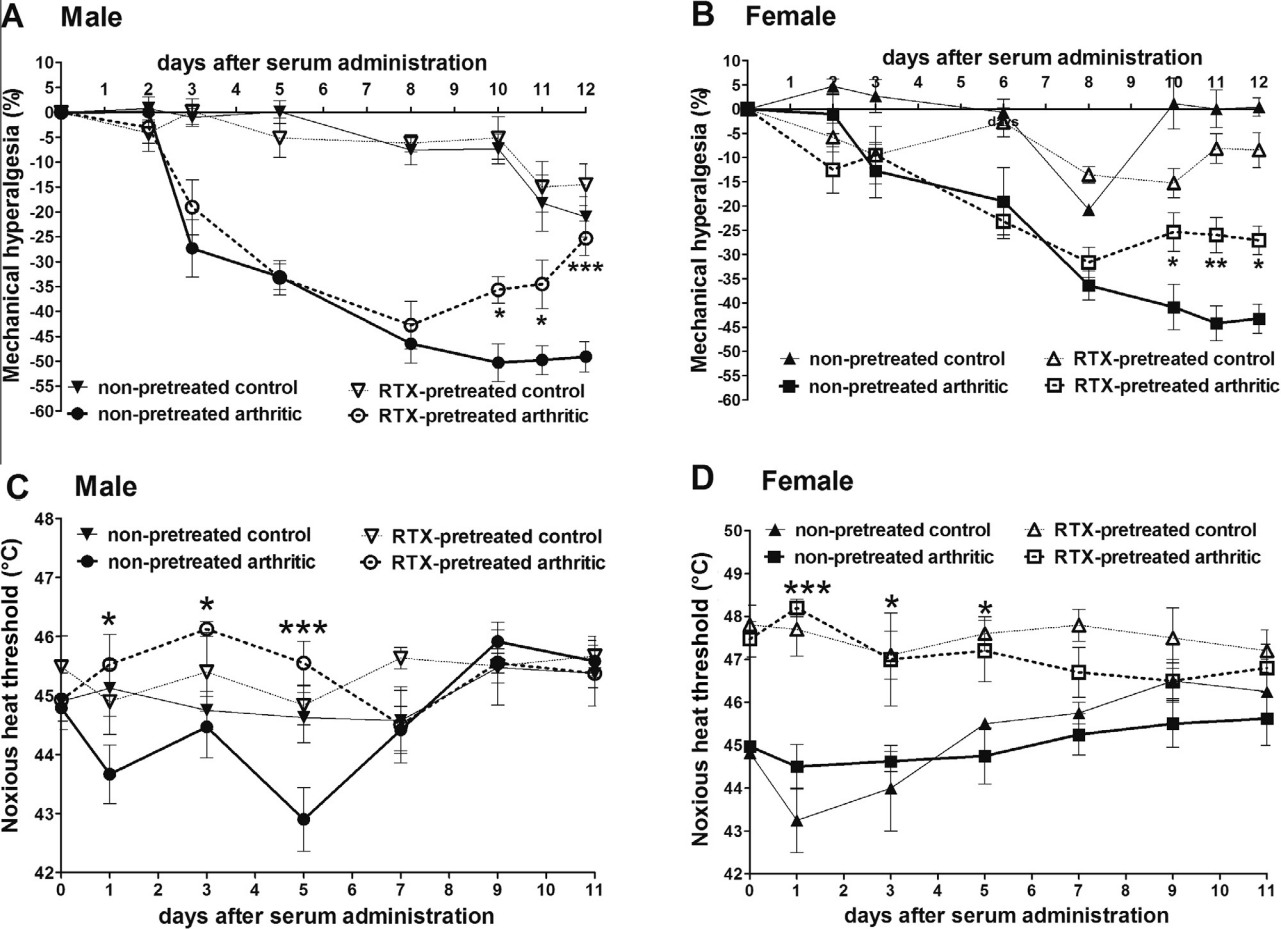
Serum-induced hyperalgesia throughout the 2 weeks experimental period. Data points represent the percentage decrease of mechanonociceptive threshold for male (A) and female (B) mice, and the absolute values of noxious heat threshold for male (C) and female animals (D) (*n* = 4–5/control non-inflamed groups, *n* = 6–8/arthritic groups; ^∗^*p* < 0.05, ^∗∗^*p* < 0.01, ^∗∗∗^*p* < 0.001 vs. non-pretreated; two-way ANOVA + Bonferroni’s modified *t*-test).

Mechanical hyperalgesia (nociceptive threshold decrease) in non-pretreated arthritic mice reached an approximately 25–30% after 5 days, which further increased to 45% by days 10 in both male and female mice (Fig. 2A and B). Significant reduction of mechanical hyperalgesia was measured in RTX-desensitized animals from day 10. Despite the development of mechanical hyperalgesia, the noxious heat threshold was not influenced by the arthritis. However, the thermonociceptive threshold of RTX-pretreated animals was significantly higher compared to mice without pretreatment between days 1 and 5 (Fig. 2C and D).

### 3.3 Similar weight loss and impaired joint function in non-desensitized and RTX-desensitized mice

Arthritis resulted in a 10–15% weight loss and 50% decrease of time spent on the grid by days 4 and 5, respectively, both in the non-pretreated and RTX-pretreated groups (Supplementary data).

### 3.4 Greater neutrophil-activity in desensitized mice in the acute arthritis phase

**Fig. 3:**
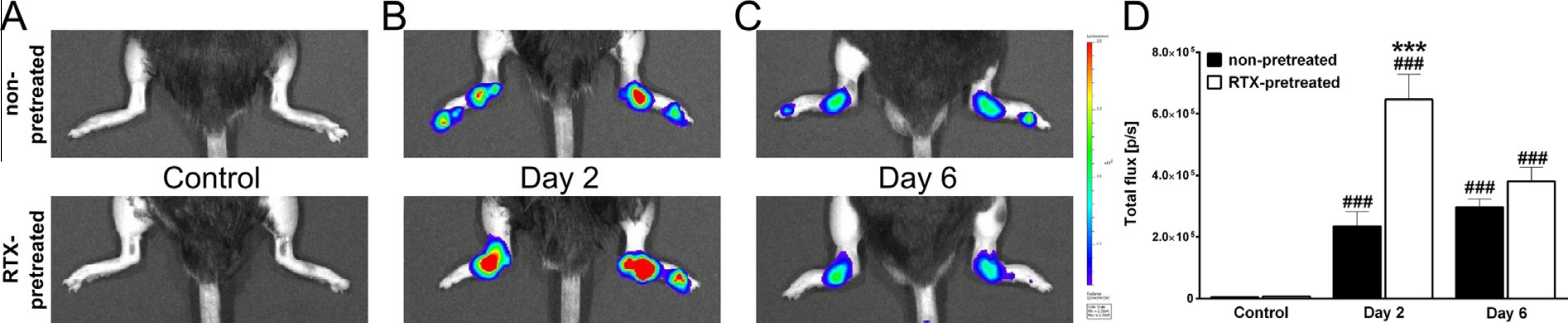
Change of neutrophil-derived myeloperoxidase activity. Panel (A) shows representative pretreatment control images, whereas panels (B and C) demonstrate inflammatory neutrophil activity on day 2 and 6 following arthritis induction, respectively. (D) Quantification of luminol bioluminescence in the diseased ankle joints. (*n* = 6–8 male mice/group, ^###^*p* < 0.001 vs. controls, ^∗∗∗^*p* < 0.001 vs. non-pretreated; Student *t*-test).

Luminol-BLI revealed a remarkable increase in neutrophil-derived MPO activity in the arthritic ankle joints of both groups, being significantly higher in RTX-pretreated mice in the early phase (day 2). This difference ceased during the later phase by day 6 (Fig. 3).

### 3.5 Increased MMP activity in RTX-pretreated animals

**Fig. 4:**
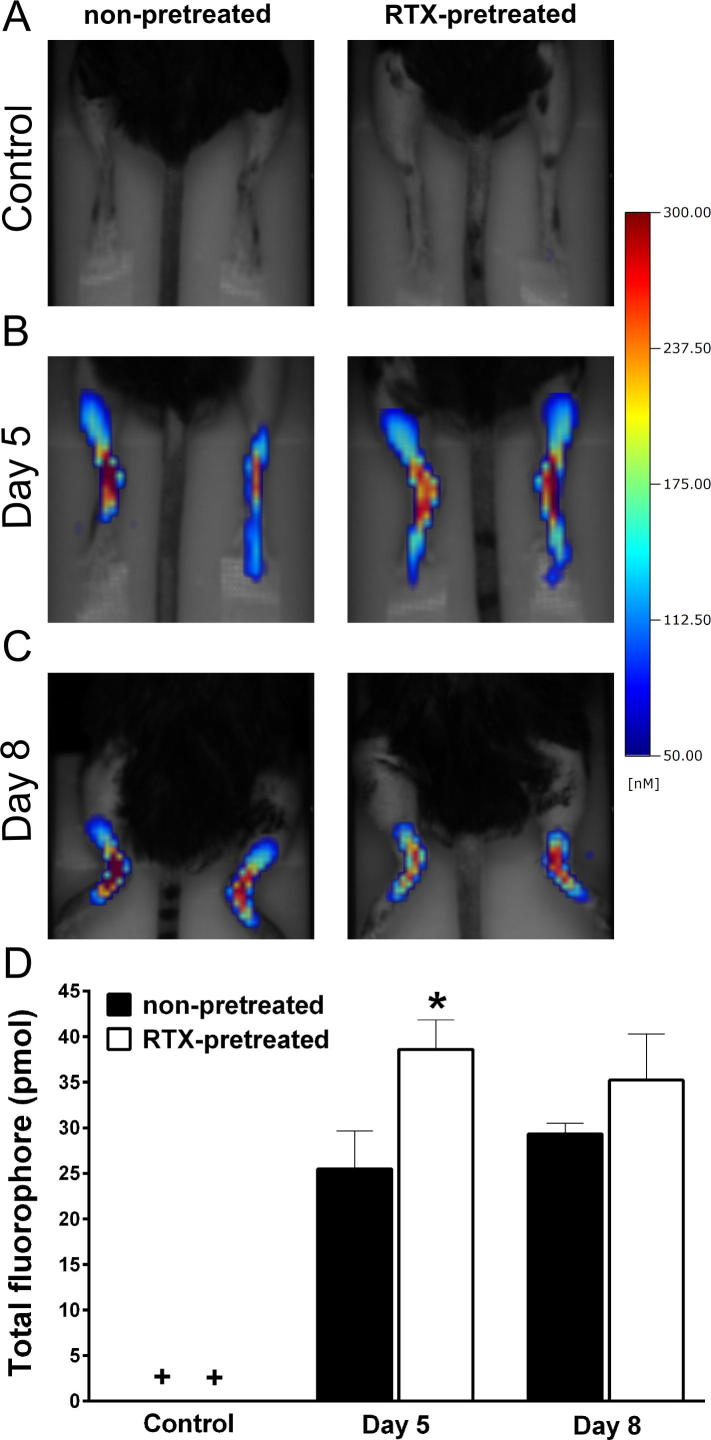
Matrix-metalloproteinase activity in the diseased hind limbs. (A) Representative pretreatment control images, (B and C) demonstrate inflammatory matrix-metalloproteinase activity on day 5 and 8 following arthritis induction. (D) Quantification the amount of fluorophore in the inflamed ankle joints (*n* = 3–4 male mice/group, ^∗^*p* < 0.05 vs. non-pretreated, + indicates that the probe was tested in intact mice in a self-control manner before the induction of the inflammation, but remained below the detection threshold; Student *t*-test).

Fluorescent molecular tomography revealed that a considerable increase in MMP activity occurred in the inflamed ankle joints of arthritic animals similarly on days 5 and 8, but no signal could be detected in intact, non-inflamed mice. MMP activity significantly enhanced after functional impairment of the capsaicin-sensitive afferents on day 5 when the differences in swelling and arthritis severity scores of the two groups were the greatest (Fig. 4).

### 3.6 Altered inflammation-induced structural changes in the bone of RTX-pretreated female mice

There were considerable differences on the micro-CT scans between bone volume/total volume ration of male and female mice under normal, intact conditions, the basal bone mass in both the tibiotarsal and distal tibial regions was lower in the female group as compared to age-matched males.

**Fig. 5:**
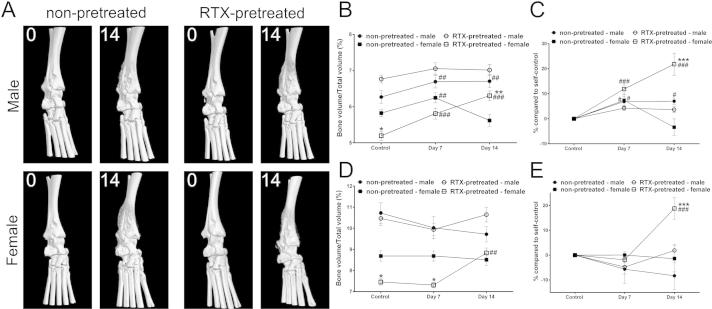
Bone structural changes in the inflamed region. (A) Representative micro-CT images of the same mice, in intact state and on day 14. (B and C) Bone volume/total volume ratio in the ankle joint, expressed as raw data and as percentage of the initial self-controls. (D and E) Bone volume/total volume ratio in the distal tibia, expressed as raw data and as percentage of the initial self-controls (*n* = 6/group, ^#^*p* < 0.05, ^##^*p* < 0.01, ^###^*p* < 0.001 vs. controls, ^∗∗^p < 0.01, ^∗∗∗^p < 0.001 vs. non-pretreated; two-way ANOVA + Dunnett and Tukey post-tests).

RTX-desensitization alone did not induce any change in the bone mass in male animals, it evoked a moderate, but significant decrease in females. Self-control quantitative analysis of the bone structure revealed minimal, but statistically significant increase in the ankle joint of non-pretreated both males and females already on day 7 due to pathological new bone formation, which was not observed in the distal tibia. Meanwhile, in RTX-pretreated arthritic females bone mass gradually and significantly increased in the ankle and the tibia reaching a remarkable, 20% gain by day 14 compared to the initial control values of the same animals (Fig. 5).

### 3.7 More severe arthritic histopathological alterations after RTX pretreatment

**Fig. 6:**
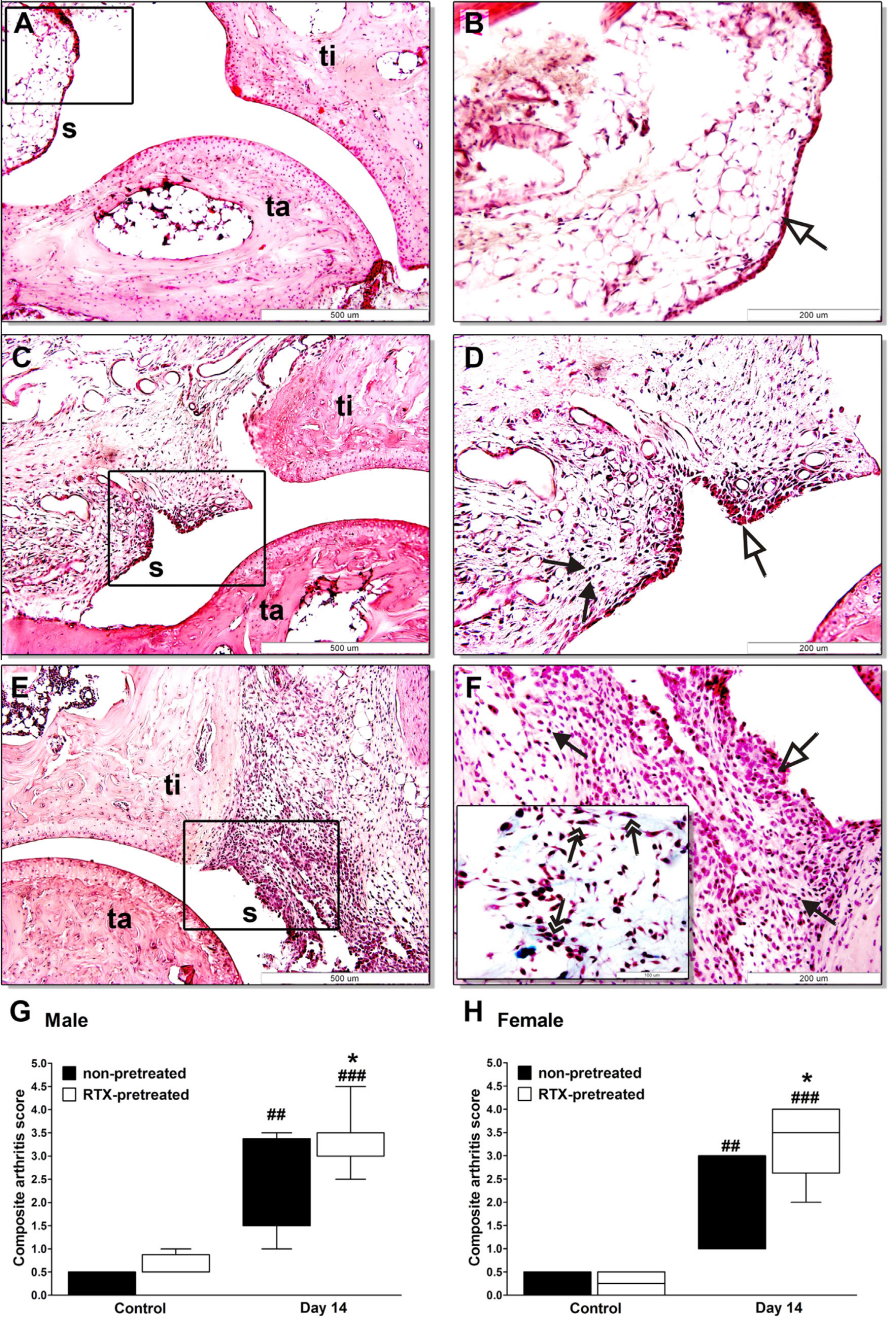
Histopathological changes of the ankle joints. Panels (A and B) show representative histopathological pictures of an intact tibiotarsal joint (ti: tibia, ta: tarsus, s: synovium), panels (C and D) demonstrate the joint structure of a non-pretreated mouse on day 14 after arthritogenic serum administration, panels (E and F) show the significantly pronounced arthritic changes of RTX-pretreated animals. (G and H) Semiquantitative histopathological scoring on the basis of synovial enlargement (white arrows), inflammatory cell accumulation (black arrows), fibroblast formation with collagen deposition (two headed arrows). Box plots represent the composite scores for male and female animals (*n* = 4–5/control non-inflamed groups, *n* = 6–8/arthritic groups; ^##^*p* < 0.01, ^###^*p* < 0.001 vs. controls, ^∗^*p* < 0.05 vs. non-pretreated; Kruskal–Wallis followed by Dunn’s post-test).

There was no histopathological difference between the intact joints of untreated and RTX-pretreated animals in either males or females (Fig. 6A and B). In non-pretreated arthritic mice characteristic chronic arthritic changes developed by day 14, such as synovial hyperplasia with a minimal mononuclear infiltration, moderate fibroblast formation and collagen deposition (Fig. 6C and D). In desensitized arthritic animals these changes were more pronounced with significantly greater synovial swelling, higher number of fibroblasts and more collagen (Fig. 6E and F). Semiquantitative scoring of these parameters showed remarkable worsening effect of RTX pretreatment on these characteristic histopathological features in both sexes (Fig. 6G and H).

### 3.8 RTX desensitization decreases arthritis-induced elevation of somatostatin-LI in the tissue homogenates

Somatostatin-LI significantly increased to 75.54 ± 3.07 fmol/g wet tissue in the arthritic paws of non-pretreated mice compared to their intact controls (25.19 ± 1.53 fmol/g wet tissue), while in RTX-desensitized arthritic animals its inflammation-induced elevation was significantly smaller, from 28.43 ± 1.19 to 62.39 ± 2.58 fmol/g wet tissue (*p* = 0.0059; Student’s *t*-test for unpaired comparisons).

## 4 Discussion

We provided here the first evidence that capsaicin-sensitive peptidergic sensory nerves play an important and complex regulatory role in a primarily autoimmune arthritis model of the mouse. Inactivation of these fibers results in significantly more severe characteristics of arthritis, such as increased swelling, MMP-activities and ROS production, inflammatory cell accumulation and histopathological alterations, but despite the enhanced inflammation decreased late mechanical hyperalgesia (Table 1.).

Peptidergic afferents densely innervate the synovium and the joint capsule and are involved in the pathophysiology of RA through the release of sensory neuropeptides and consequent modulation of cytokine production (Konttinen et al., 2006). Increasing evidence suggests that modulating the function of these nerves might open new perspectives in arthritis therapy (Helyes et al., 2004; Szabó et al., 2005). The present results are perfectly supported by our revolutionary findings obtained in the adjuvant arthritis rat model 10 years ago, when we described a potent protective function of capsaicin-sensitive afferents via somatostatin release (Helyes et al., 2004). However, that time we had no experimental tools to have a deeper insight into the underlying mechanisms. Our optical *in vivo* imaging methods provide a great opportunity to investigate the cellular components of the arthritic process and provide direct evidence for the importance of sensory–immune interactions. Activation of the capsaicin-sensitive afferents inhibits both MPO and MMP activities, decreases leukocyte activity, and interestingly, in females even attenuates pathological new bone formation. Although there was no difference between male and female mice in any inflammatory parameters, our unique finding obtained by quantification of the self-control micro-CT scans is that there was in fact a decreased bone mass in females compared to age-matched males. Furthermore, in females inactivation of the capsaicin-sensitive afferents resulted in basically decreased bone volume, but the arthritis-induced pathological bone formation was more severe. These results are supported by recent data showing remarkably lower BV/TV morphological parameter and higher histopathological osteophyte score in old female C57Bl/6 mice compared to the age-matched males (Cai et al., 2014). Additionally, the same BV/TV parameter determined in the human radius also provided similar results; this value was lower in all age groups of women compared to men (Vanderschueren et al., 2014). Since unlike in female mice, in RTX-pretreated males we could not detect a significantly increased pathological bone formation as compared to the non-pretreated animals, it can be suggested that the androgens might have a protective role on the bones particularly under inflammatory conditions. It is clear that sex steroids are important influencing factors in osteoclast/osteocyte/chondrocyte functions and that in men not only the bone mineral density, but also the bone structure (bone length, width and rigidity) differs from these in women (Vanderschueren et al., 2014). However, the precise mechanisms of peptidergic sensory nerve activation and bone turnover regulation are still unclear, because there are very few data about the importance of afferents/TRP channels in chondrocyte–osteoclast–osteoblast functions. Desensitization itself is reported to affect only the nerve-endings and not the non-neural TRPV1 channels (Czikora et al., 2013; Kun et al., 2012; Bíró et al., 1998), but influencing the endovanilloid/endocannabinoid system in chondro- and osteocytes can be involved in bone formation and resorption, although these data are available only on osteoclasts from osteoporosis patients (Rossi et al., 2011).

It is well-known that in the early stage of the inflammatory reaction in this model, the activation of neutrophils is a predominant component (Bevaart et al., 2010) and MPO is the major constituent of neutrophil azurophilic granules. Moreover, it is recently shown that in patients with active rheumatoid arthritis a very high concentration of MPO can be detected and it positively correlates with IgM levels (Wang et al., 2014). Similarly to what we found in this arthritis model, elevated MPO-levels in RTX-desensitized mice were previously detected in LPS-induced acute airway inflammation (Elekes et al., 2007). Our MMP results are also in good correlation with sporadic earlier evidence demonstrating that TRPV1 receptor activation results in decreased MMP-9-secretion (Tauber et al., 2012). Both MPO and MMP are important, but not the exclusive participants of this complex inflammatory process, and their correlation with either edema formation, pain or bone pathophysiology is only indirect. Although we do not have data obtained from later phase of the model, the activation of these enzymes leads to a variety of pathophysiological alterations including the recruitment of different inflammatory cells, as well as activation of other enzymes (e.g. NOS; Arnhold and Flemmig, 2010), which can result in a propagation of the inflammatory processes or even deformation of bones, despite lower MPO or MMP levels.

To understand the mechanisms involved in the inhibitory action of capsaicin-sensitive afferents, somatostatin was determined in the tibiotarsal joint homogenates. Somatostatin and its receptors (sst_1__–__5_) are widely distributed throughout the body and show a prominent expression in the sensory nerve endings (Pintér et al., 2006), but they are also present on immune cells suggesting its important regulatory function in inflammatory diseases (Pintér et al., 2014). Somatostatin ameliorates RA symptoms not only in murine models (Imhof et al., 2011), but also as a chronic intra-articular treatment in humans (Fioravanti et al., 1995; Paran et al., 2001). We found that it was increased in the arthritic joints, but significantly decreased after RTX-pretreatment. Therefore, its inflammation-evoked elevation is likely to be derived from the capsaicin-sensitive fibers, and be involved the protective actions of these nerves. The MMP-activity increase in desensitized mice is also consistent with these results, since somatostatin was reported to reduce MMP-1, -2 and -9 mRNA expression and MMP-1 production by synovial cells of RA patients (Takeba et al., 1997).

Intriguingly, in contrast to our earlier findings in RTX-pretreated rats in the adjuvant arthritis model (Helyes et al., 2004), the increased inflammation was not accompanied by a proportionally enhanced mechanical hyperalgesia. In the late phase, when inflammatory signs were attenuated, but mechanical hyperalgesia was still present, it was even significantly milder in RTX-pretreated mice. Compared to the greater severity of inflammation, mechanical hyperalgesia is clearly smaller in the RTX-pretreated group. Therefore, capsaicin-sensitive nerves might participate in arthritic mechanical hyperalgesia during the whole process, but the difference was only manifest when the degree of inflammation was equal in both groups. Capsaicin-sensitive nociceptors are polymodal, i.e. activated by noxious heat, chemical and mechanical stimuli, and their role is proven *in vivo* in thermonociception (Almási et al., 2003; Cavanaugh et al., 2009; Mishra and Hoon, 2010; Danigo et al., 2014; Bölcskei et al., 2010). In contrast, mechanonociceptive thresholds were not different in desensitized mice if von Frey filaments were used (Cavanaugh et al., 2009; Mishra and Hoon, 2010), but increased mechanical thresholds were found with the Randall–Selitto pressure test (Danigo et al., 2014). It is likely, that the latter method, as well as our aesthesiometer activated a different mechanonociceptive neurone subset, which explains the difference after RTX pretreatment. Furthermore, recent data showed that in the chronic phase of the K/BxN arthritis, when swelling and hyperemia disappeared, neuropathic pain developed. It was only relieved by gabapentin, a typical adjuvant analgesic for neuropathy (Christianson et al., 2010). In heat and mechanical hyperalgesia TRPV1 channels are the most important participants (Sousa-Valente et al., 2014; Hulse et al., 2014). In agreement with these results we detected a significantly attenuated mechanical hyperalgesia in RTX-pretreated animals only in the late phase (from day 10). This strongly suggests that TRPV1 channels play a pivotal role in pain mediation, as described in neuropathy (Brito et al., 2014) and arthritis models (Fernandes et al., 2011; Kelly et al., 2013), but the time course-dependent involvement of these channels has never been proven. Thermal hyperalgesia is neither characteristic in K/BxN arthritis, nor in RA patients (Edwards et al., 2009; Leffler et al., 2002). However, we found a significant difference in the RTX-pretreated group during the 1st week, which is probably due to the well-known heat threshold increasing effect of RTX (Almási et al., 2003), but arthritis did not alter thermosensitivity.

Based on all these data capsaicin-sensitive peptidergic sensory nerves and sensory–immune interactions are important regulators of immune-mediated arthritis. Their activation inhibits the characteristic arthritis symptoms (edema, inflammatory cell activation and functions) at least partially through somatostatin release, but despite this potent anti-inflammatory role, they mediate the later pain response. These results are in agreement with our previous results obtained from LPS-induced pneumonitis, where the inactivation of the capsaicin-sensitive nerves leads to greater inflammation (cell accumulation, edema), but decreased bronchial hyperreactivity (Elekes et al., 2007). In contrast, it has very recently been published that both RTX-desensitization and TRPV1 receptor deficiency ameliorate the clinical severity in the IL-23-mediated, T-cell dependent psoriasiform dermatitis model (Riol-Blanco et al., 2014).

It can be concluded that neuropeptide-containing sensory nerves exert a complex regulatory function in inflammatory conditions, and the overall effect of their activation depend on the tissues and the pathophysiological mechanisms of the disease.

## Figures and Tables

**Supplementary Fig. 1:**
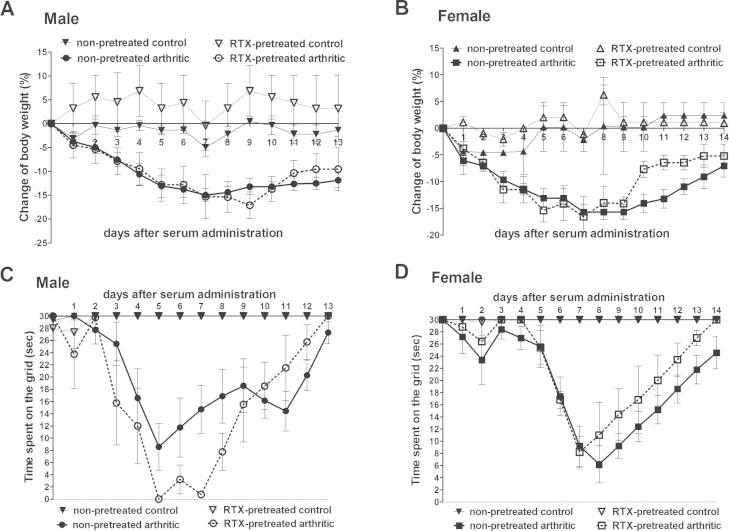
*Serum-induced weight loss and impaired joint function:* data points represent the percentage change of weight loss of male (A) and female (B) mice compared to the initial control values and the absolute values of time spent on the grid for male (C) and female animals (D) (*n* = 4–5/control groups, *n* = 6–8/arthritic groups; two-way ANOVA + Bonferroni’s modified *t*-test).

**Table 1:** Summary of functional, morphological and immunological alterations in RTX-pretreated mice compared to the non-pretreated animals. Edema formation, arthritis score, neutrophil activity, matrix-metalloproteinase activity and histopathological changes were significantly aggravated, while paw somatostatin level and mechanical hyperalgesia were significantly attenuated in RTX-pretreated animals. Noxious heat threshold, weight loss and joint function did not differ between the two groups.

Arthritis parameters	Effect of RTX-pretreatment on arthritis changes
Paw edema	↑
Arthritis score	↑
Neutrophil-activity	↑
Matrix-metalloproteinase activity	↑
Pathological bone formation (in females)	↑
Histopathological changes	↑
Mechanical hyperalgesia	↓
Paw somatostatin level	↓
Heat threshold	–
Joint function	–
Weight loss	–
